# Union

**Published:** 1894-11

**Authors:** 


### EDITORIAL.

### Union.

There are in the United States two associations, each claiming in some respects national character. The one, the American Dental Association, was formed by delegates from existing local and State societies; this is the only channel for the continuation and support of its membership. It was organized in 1859. The other, the Southern Dental Association, consists of a membership that is brought in through a membership committee. Any reputable member of the profession, upon application to this membership committee, may be received regardless of membership in any other society.

The question has been frequently asked in past years as to whether the interest of the profession as a whole is best subserved by the continuance of these two bodies. In the early years of these associations circumstances did not seem to be as favorable for their union as now exist. The condition of these two bodies is so changed within the last few years, by holding combined sessions and by interchange of membership, that now no good reason exists for the separate organizations. Doubtless, the consensus and indeed the wish of a large number of the membership of both bodies is that a union should be effected. A movement has already been made looking to this result, and it is earnestly to be hoped that the matter will be taken up by those having it in charge and carried to a successful conclusion. There is no apparent reason why this division should longer exist; it certainly is a waste of power and resource. The profession is homogeneous throughout our country, and the representative body of the dental profession should have its membership from the profession in all parts of the country. If ever any jealousy or antagonism existed between these two bodies there is certainly now nothing of these extant. Many have membership in both bodies. Many dentists of the north have membership in the Southern Dental Association and are in almost constant attendance upon its annual gatherings; the same is true in regard to Southern dentists in respect to the American Dental Association, and to such an extent is this true that it seems unreasonable and against the best interests of the profession that this separation should be longer continued, and we most urgently request those to whom the matter was specially committed that they move forward in their efforts for this accomplishment as promptly as circumstances will at all permit.

